# Core knowledge translation competencies: a scoping review

**DOI:** 10.1186/s12913-018-3314-4

**Published:** 2018-06-27

**Authors:** Anastasia A. Mallidou, Pat Atherton, Liza Chan, Noreen Frisch, Stephanie Glegg, Gayle Scarrow

**Affiliations:** 1 School of Nursing, University of Victoria, B236 – HSD Building, 3800 Finnerty Road (Ring Road), Victoria, BC V8P 5C2 Canada; 2 School of Nursing, University of Victoria, PO Box 1700 STN CSC, Victoria, BC V8W 2Y2 Canada; 3 Alberta Innovates – Health Solutions & University of Alberta, 1500, 10104 103 Ave, Edmonton, AB T5J 4A7 Canada; 4 Sunny Hill Health Centre for Children, 3644 Slocan Street, Vancouver, BC V5M 3E8 Canada; 5 Michael Smith Foundation for Health Research, 200 - 1285 West Broadway, Vancouver, BC V6H 3X8 Canada

**Keywords:** Competencies (attitudes, Knowledge, Skills), Evidence-based practice, Knowledge brokering, Knowledge translation, Knowledge utilization, Professional competencies, Scoping review

## Abstract

**Background:**

Knowledge translation (KT) is the broad range of activities aimed at supporting the use of research findings leading to evidence-based practice (EBP) and policy. Recommendations have been made that capacity building efforts be established to support individuals to enact KT. In this study, we summarized existing knowledge on KT competencies to provide a foundation for such capacity building efforts and to inform policy and research. Our research questions were “*What are the core KT competencies needed in the health sector?”* and *“What are the interventions and strategies to teach and reinforce those competencies?*”

**Methods:**

We used a scoping review approach and an integrated KT process by involving an Advisory Group of diverse stakeholders. We searched seven health and interdisciplinary electronic databases and grey literature sources for materials published from 2003 to 2017 in English language only. Empirical and theoretical publications in health that examined KT competencies were retrieved, reviewed, and synthesized.

**Results:**

Overall, 1171 publications were retrieved; 137 were fully reviewed; and 15 empirical and six conceptual academic, and 52 grey literature publications were included and synthesized in this scoping review. From both the academic and grey literature, we categorized 19 KT core competencies into knowledge, skills, or attitudes; and identified commonly used interventions and strategies to enhance KT competencies such as education, organizational support and hands-on training.

**Conclusions:**

These initial core KT competencies for individuals provide implications for education, policy, knowledge brokering, and future research, and on the need for future evaluation of the KT competencies presented. We also discuss the essential role of organizational support and culture for successful KT activities/practice.

**Electronic supplementary material:**

The online version of this article (10.1186/s12913-018-3314-4) contains supplementary material, which is available to authorized users.

## Background

Despite the exponential growth of publications on knowledge translation (KT) in health-related disciplines, a significant gap exists between “what is known” (evidence) and “what is done” (practice) at all levels of decision-making to improve health [1]. This gap may be costly in three ways: premature adoption or overuse of treatments, delivery of sub-optimal or unnecessary care, and the initiation of new studies that may not be informed by the latest research findings or that may not fully address the needs of knowledge users [2–6]. KT is the broad range of activities meant to improve the use of health research in practice and to inform further research leading to evidence-based decision-making in healthcare. Internationally, various terms are used, sometimes interchangeably, to describe these activities including knowledge utilization, research uptake, knowledge mobilization, research utilization, research to action, dissemination and implementation [1, 7, 8]. In this scoping review, we use the term “KT” as it is officially defined by the Canadian Institutes of Health Research (CIHR) [9], “a dynamic and iterative process that includes synthesis, dissemination, exchange and ethically-sound application of knowledge to improve the health of Canadians, provide more effective health services and products and strengthen the health care system”, as occurring within a complex system of interactions between knowledge producers and knowledge users that may vary in intensity, complexity and level of engagement depending on the nature of research and the needs of knowledge users [10]. Four elements of KT are emphasized in this definition: synthesis, dissemination, exchange, and application of knowledge. While multiple efforts to support KT activities have been enacted [11], these have not been completely successful [12, 13]. Several factors [14] negatively influencing the use of research findings have been suggested; these include limited efforts for building individuals’ capacity to engage in KT, clinician negative attitudes, resistance to change, time constraints and limited resources [15, 16]. Further, specific competencies on which to build KT capacity have yet to be identified [17, 18]. Nonetheless, there is agreement that capacity building efforts targeting individuals, teams, organizations, and systems should be incorporated into each stage of the KT process [19]. The focus of our project is building capacity in research use at the individual level. Therefore, we have conducted a scoping review on competencies needed by individuals to engage in KT and, based on this review, we compiled a series of core competencies to support KT for individuals working in the health sector. Incorporation of these KT competencies into education, job expectations, and performance appraisals may positively influence consistency and quality of healthcare and reduce healthcare system expenses [12]. Definitions of the main concepts and terms used in this paper are described elsewhere [20].

### Purpose

To summarize existing knowledge on competencies needed to enact KT. Our research questions were “*What are the core KT competencies for those in the health sector and what are the interventions and strategies to teach and reinforce those competencies?*” The main objectives were to:

- Systematically explore the relevant theoretical/conceptual, empirical and grey literature on KT competencies. Usually, a scoping review includes both academic and grey literature in order to detect relevant themes on the topic.
- Map the publications by identifying key themes for each group of KT competencies.
- Record strategies for teaching, improving and supporting these competencies while identifying research gaps in our knowledge about building KT capacity.
- Summarize and disseminate review findings to stakeholder groups in relevant fields (e.g., nursing, medicine, rehabilitation, health policy) for the purposes of designing future studies and systematic reviews.

In comparison with the protocol of this scoping review [20], the actual review process has been modified. Specifically, we amended the purpose of the review by focusing only on

- KT competencies in general; not those for three discrete audiences such as knowledge users, knowledge brokers, and knowledge producers/researchers. The main reason for this modification was that the relevant literature has not been categorized that way. We have not found any publication that referred to KT competencies per each of these stakeholder groups.
- The primary research question “What are the core KT competencies in the health sector and the interventions and/or strategies to teach and reinforce those competencies?” We also excluded the last objective about the development of self-assessment tools (the KT Pathways) for professional development of those three target audiences (i.e., knowledge users, knowledge brokers, and knowledge producers/researchers).

## Methods

To address the objectives above, we used the methodological approach for scoping reviews described by Arksey and O’Malley [21] that includes five stages 1) identifying relevant publications; 2) selecting the literature; 3) charting the literature; 4) synthesizing and summarizing the findings; and 5) reporting the results. Throughout the process, we involved and consulted members of a formal Advisory Group to incorporate various perspectives, enhance our understandings of the literature and to ensure the applicability of our review findings. The Advisory Group consisted of 13 experts such as KT and knowledge mobilization consultants; physical therapy KT broker; KT implementation scientist; member of Arthritis Patient Advisory Board & a scientist in Arthritis Research Canada; director in academic development of Provincial Health Services Authority; regional practice leader in research & KT; librarian on research in KT; associate director in KT of Alberta SPOR SUPPORT Unit; academic researchers; and the president & CEO of MSFHR (for details, please see Acknowledgments). We have not registered this scoping review with PROSPERO, because scoping reviews are not usually registered; however, the review protocol has been published [20].

### Searches (identifying relevant publications)

Targeted *search strategies* were initially developed in consultation with our team’s librarian. The literature *search terms* included controlled vocabulary and various keywords related to the KT field such as KT, knowledge utilization/use, research use; and competencies related to KT in the health sector. Because of the lack of appropriate subject headings and a large amount of related but not on-topic literature, the search strategy focused on identifying key terms in titles and abstracts to more efficiently target results for which KT competencies were the primary focus. Our *search sources and strategies*, presented in Additional file 1, included a) health, healthcare and interdisciplinary electronic databases; b) grey literature sources; c) hand searching of relevant specialized key journals; d) reference lists in publications identified in (a), (b) and (c); and e) works identified through personal contacts of the working group and stakeholder groups. Search limits were applied in *language* (English only) and *publication date* (between January 2003 and November, 2017). The publication date restrictions reflect the developing interest in the KT field since the 1990s [8, 22] and the exponential growth in publications on KT after 2000s [8]. Search results were imported into a bibliographic manager (i.e., Mendeley) and duplicates removed.

### Study inclusion and exclusion criteria (selecting the literature)

*Inclusion*: All empirical and theoretical/conceptual peer-reviewed publications and grey literature in health that examined KT competencies were considered for inclusion. Specifically, each publication had to:

- Be an empirical or theoretical/conceptual peer-reviewed article or grey literature in health;
- Include both concepts and/or sub-concepts of *knowledge translation* or any other similar term (e.g., knowledge utilization, knowledge use, knowledge transfer) and *competency* (i.e., knowledge, skills, attitudes) or any component of competencies specifically related to KT competencies; and
- Have an abstract and purpose clearly stated (for empirical and conceptual publications only); grey literature was reviewed in the absence of an abstract or purpose.

#### Exclusion

Publications written in non-English languages and those published before 2003 were excluded. Restrictions according to status of publication (e.g., in review, accepted, in press) were not applied.

### Selection and classification of literature (charting the literature)

In this iterative process, we retrieved and reviewed search results using the predetermined inclusion and exclusion criteria. Three groups of two or three reviewers independently screened titles and abstracts of all publications retrieved. Publications identified as potentially relevant were retrieved in full text and reviewed. We resolved discrepancies regarding a publication inclusion through discussion and consensus among all reviewers. We classified retrieved publications into research, theoretical/conceptual publications, and grey literature using a data extraction instrument developed for this study purposes. At least two reviewers independently extracted data from a sample of ten publications to determine the consistency of their approach with the purpose of the review. We discussed and resolved discrepancies in data extraction by consensus.

### Data analysis and synthesis (synthesizing and summarizing the findings)

We carried out standardized steps to analyze and synthesize the data. In particular, we recorded the data related to the origin of study, type of publication, purpose, abstract, use of theoretical framework, study design, study population, KT competencies used and defined, measurement tools, strategies to improve KT competencies (e.g., type of interventions), and results relevant to KT competencies. Theoretical and empirical literature was summarized as a traditional integrative review [23]. We summarized publications and their characteristics (e.g., type of publication, theoretical frameworks, study design, intervention) in creating a literature map. We followed a similar process for the grey literature; we identified commonalities, and extracted both competencies discussed and strategies suggested to build competencies. The findings from the empirical studies and theoretical and grey literature were synthesized and are presented below as a narrative review [24].

## Results

### Publications retrieved

**Fig. 1:**
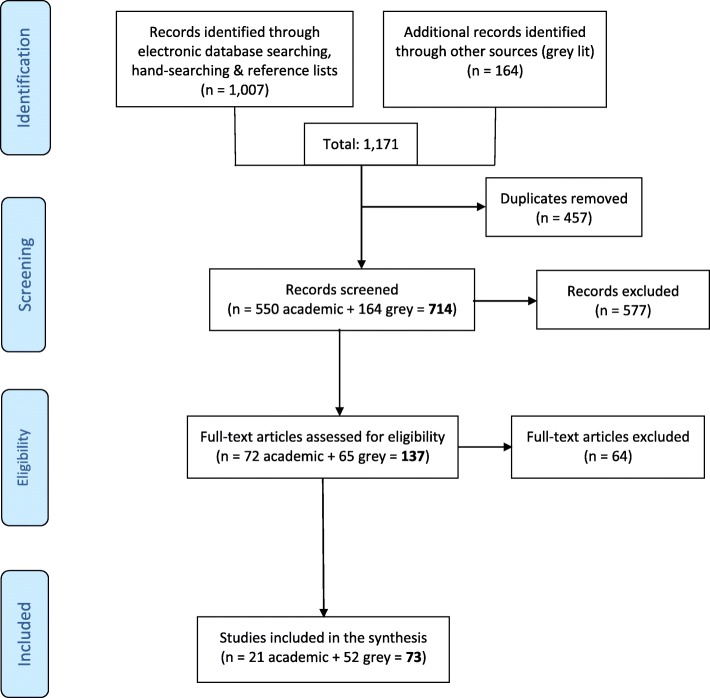
Literature Search Process

Search strategies revealed 1171 academic and 164 grey literature. After removing duplicates, 550 academic and 164 grey publications remained for the preliminary screening against the predetermined inclusion/exclusion criteria; and 72 academic and 65 grey publications for the full-text review. Only 15 empirical and six conceptual academic, and 52 grey publications met the eligible criteria and were included in the review (Fig. 1). The findings from the academic and grey literature are described separately but grouped together to identify KT competencies relevant to both literature sources. The following section is organized accordingly with descriptions of the peer-reviewed publications first, followed by the grey literature resources. Next, we present a set of 19 KT competencies that are the synthesis of both the peer-reviewed and the grey literature.

A. Peer-Reviewed/Academic Primary Publications

Included peer-reviewed publications were published since 2003 in Canada or the USA (15 of 21; 71.43%) or elsewhere (i.e., Australia, Kenya, Saudi Arabia, Sweden, UK), and classified into the following categories (Additional file 2):

1. *Empirical (* i.e. *, research projects or studies)*

All included studies had a descriptive and/or exploratory design (e.g., cross-sectional surveys, interviews, case studies) reflecting the infancy of research on this topic. Quantitative studies [25–29] examined individual professional self-reported perceptions of either own competency levels or their backgrounds in EBP and primarily used surveys to obtain data. Qualitative studies [30–37] had varied designs: participatory action research [30, 35]; case study report [31]; evaluation of educational programs [32, 33]; interviews [36], concept mapping [37], and a workshop description on applying innovative methods for capacity building in KT [34]. Mixed methods studies [38, 39] included a program evaluation using multiple data sources [38] and a second study using an explanatory longitudinal design [39].

2. *Theoretical/conceptual*

Six theoretical/conceptual publications were included. Three were discussion papers providing recommendations relevant to KT activities and capacity development [5, 40, 41]; and three other publications on knowledge brokering in Canada [43–45].

### Theoretical frameworks/models/theories

The Knowledge to Action framework [34, 35, 44], Diffusion of Innovations Theory [25], Utilization-Focused approach [30], and the Promoting Action on Research Implementation in Health Services (PARiHS) framework [38] were used as a foundation supporting the included studies (Additional file 3).

### Populations, samples, measurement instruments, and findings

Registered nurses were the most frequently studied population [25–27, 32] followed by registered chiropractors [28]; educators and administrators [25, 33, 36]; family medicine consultants [34], managers in healthcare organizations [30, 31], occupational therapists [35], and researchers and policy-makers [37] (briefly described in Additional file 2). No study reported any measurement instrument to assess KT competencies.

### Interventions/strategies to improve KT competencies

We identified only a few interventions and strategies to enhance KT competencies. The most popular (described in Additional file 2) included

- Formal and continuing education [25, 26, 32], and
- Organizational support [30] such as time to review studies, dialogue between administration and staff, creation of a culture where EBP is valued and expected [26], and active communication [31].

B. Grey Literature

### Origin and type of publications

Most of the included grey literature originated in Canada and the USA (47 of 52; 90.4%), while five of 52 (9.6%) were from elsewhere (i.e., Australia, England, Scotland, Switzerland). We grouped these resources into two categories: job descriptions and other documents (Additional file 4).

1. *Job descriptions*

Twenty three job descriptions revealed a wide variety of job titles, minimum required education levels and years of experience. The most common job title was that of knowledge broker [46–51] followed by research associate [52–54], KT manager [55, 56], program coordinator [57–60], KT director [61, 62], KT specialist [63, 64], knowledge mobilization coordinator [65], KT officer [66], KT and policy manager [67], and postdoctoral fellow [68]. A master’s degree was the most frequently required minimum education requirement noted (*n* = 17) followed by a bachelor (*n* = 4) and doctoral degree (*n* = 2). The minimum required related-KT experience ranged from none (*n* = 7) to more than seven years of experience (*n* = 3).

2. *Other documents*

Twenty nine additional documents included KT guides [69–80], tools [81–84], frameworks and theories [85–88], models [89, 90], online learning modules [91, 92], and other resources such as a policy link [93], an environmental scan [94], templates [95, 96], and a workshop report [45].

### Interventions and strategies to improve KT competencies

Drawn primarily from the grey literature, we identified only a few interventions and strategies (e.g., hands-on training) [42], and suggestions for interventions to improve and expand KT competencies, the majority of which refer to educational sessions and strategies [97, 98] as well as to leadership and communication strategies [35], or funding a knowledge translation champion one day per week [35].

C. KT Competencies

Findings from both the academic and grey literature are summarized as 19 core KT competencies (Table 1), which are grouped in three categories (i.e., knowledge, skills, attitudes) and discussed below. In this work, we do not claim that each competency is independent from another. We attempt to disentangle and analytically described the identified competencies, in order to provide lists of the components of the competencies. The reader needs to take into consideration the complexity theory in cognitive psychology and that the concepts/competencies described in the paper attempt to explain the complex phenomenon of KT competencies required for KT activities, which are not explainable by traditional theories. We attempted to simplify the required competencies by breaking them down into their constituent parts. We used a structure of knowledge, skills, and attitudes to present our findings from the literature at different categories of competencies. This structure of competencies has been also used in the British Columbia (BC) Education Pathway and the Health Services Researcher Pathway (HSRP) studies. We recognize that complex behavior emerges from simple competencies, which interact as in all complex systems and networks. Following the discussion, competencies derived from the grey literature only are noted.

**Table 1:** Nineteen core KT competencies retrieved from the literature

	KT Competencies
1.	“*Knowledge*”
1.1.	Understanding the context
1.2.	Understanding the research process
1.3.	Sharing knowledge
1.4.	Being aware of evidence resources
1.5.	Understanding KT and EBP processes
1.6.	Understanding translation and dissemination activities
2.	“*Skills*”
2.1.	Collaboration and teamwork
2.2.	Leadership
2.3.	Sharing knowledge
2.4.	Knowledge synthesis
2.5.	Dissemination of research findings
2.6.	Use of research findings (or research use)
2.7.	Fostering innovation
2.8.	Knowledge brokering
3.	“*Attitudes*”
3.1.	Confidence
3.2.	Having trust
3.3.	Valuing research
3.4.	Self-directed lifelong commitment to learning
3.5.	Valuing teamwork
4.	“Other”
4.1.	Knowledge of quality improvement methods and tools, communication strategies, and health policy and systems.
4.2.	Skills related to KT planning, project management, information technology use, sound judgment, and discretion/tact/diplomacy and resourcefulness.
4.3.	Attitudes such as integrity, commitment to professional work ethic and behaviour in interaction with contacts, commitment to high standards of professionalism, and interest in the latest developments in communications.

1. *KT competencies – Knowledge*
  Competency 1.1 – *Understanding the context*: the pragmatic understanding of organizational practices and knowing “how things really work” in specific organizational settings [30] and/or local healthcare systems [50, 99]. This competency refers to the knowledge one needs to have about practical environments that affect new knowledge application [86],; the strategic health system goals of each organization or unit [93]; environments interested in and open to research [30] that apply basic rules of marketing and market dynamics [50, 99], sustainability of knowledge and research [45], and learning process and system thinking [41]; to accept the interdependence of knowledge users, practitioners, managers, support staff [87] and know how to gauge the applicability and adaptability of evidence to user contexts [82].
  Competency 1.2 – *Understanding the research process*: knowing the process of conducting research. This competency includes knowing how to form research questions, understand search strategies [29, 36, 37, 52], identify appropriate databases on a given topic [28, 70], know how to build relationships with stakeholders [79], appraise the literature and understand various research methodologies [74], as well as comprehend how evaluation research is related to research use [29, 42, 61, 100] and how research findings can influence practice [26, 30] or policy [36].
  Competency 1.3 – *Knowing how knowledge is disseminated*: understanding meaningful ways to share available and accessible knowledge/evidence [35, 79]. This competency includes understanding communication techniques such as social media [96] and how language facilitates collaborative activities [26, 30], knowing the theory and practice of group facilitation [41, 95] to support evidence-informed action, evaluate outcomes [42] and improve the decision-making process [31].
  Competency 1.4 – *Being aware of evidence resources*: knowing ways to find available resources that support organizational information [27], understand the organization and structure of electronic library databases [25], the use of evidence based tools and databases [26] (e.g., the Cochrane Collaboration operation [101]) and the role of digital, regular strategic-intelligence bulletins (listservs), blogs, social media (e.g., Facebook, Twitter), YouTube, newsletters [76] in finding research results [27, 32], writing recommendations [41], and facilitating the use of these resources [26, 102].
  Competency 1.5 – *Understanding KT and EBP processes*: knowing the KT activities relevant to clinical practice, policy-making, and research. This competency requires knowing the definitions of EBP and KT, being aware of the 5-step EBP process [102], and understanding models and theories of KT [35, 44, 86, 92, 102]. This competency includes understanding that interventions need to be feasible and practical [43, 44], needs assessments are useful for best practices [45], and understanding that there are common barriers and facilitators of EBP [102].
  Competency 1.6 – *Understanding knowledge translation and dissemination activities*: knowing how to interpret research findings for various audiences and uses. This competency includes knowing the process for conducting knowledge syntheses [45], for addressing KT questions [33], understanding templates for KT activities and dissemination/implementation models [90], knowing how to implement KT projects within organizations [44], understanding the diffusion of innovation model [41], knowing elements of the knowledge transfer process [35, 86, 89, 92, 102], understanding both end-of-grant and integrated KT activities [33, 72], and knowing how to examine determinants of knowledge use across different settings and groups [33] and how decision-makers find or commission synthesized research [85].

2. *KT competencies – Skills*
  Competency 2.1 – *Collaboration and teamwork*: ability to develop effective, authentic and respectful working relationships with peers and others [30, 36, 44, 45]. This competency requires maintaining professional relationships [36, 78, 79, 86, 95], establishing trusting relationships and engaging with others [38]. Teamwork also includes bringing people together [38], facilitating social interaction, using technology and collaborative processes for skill development, adult education [38], networking and communication [30, 36], moderating discussions and meetings [44, 45] and facilitating integrated KT [33].
  Competency 2.2 – *Leadership*: ability to scan the context, facilitate stakeholder involvement in evidence-based decision-making, and influence skill development and act upon stakeholders’ views and needs [36, 41, 87]. A leader can involve others in decision-making, support the development of skills in others [31, 41], and change problem-solving and consultancy processes [41]. This competency includes persuasive [38], personal and organisational skills [41], and verbal and written communication skills [35–38].
  Competency 2.3 – *Sharing knowledge*: ability to share information and data with diverse stakeholders. This competency includes having skills related to conducting research of relevance to intended users [30, 86] and co-creation of knowledge with stakeholders (e.g., writing research proposals for funding and developing appropriate evaluation plans [30, 88]) and the capacity to collaboratively design, guide and assess implementation of evaluations, interpret data and make data-driven decisions to promote use of evidence [30, 42].
  Competency 2.4 – *Knowledge synthesis*: having skills to combine research findings and grey literature following robust processes. This competency includes the capacity to conduct knowledge syntheses [45] and address KT questions [33]. Knowledge synthesis relies on the abilities to form research questions, develop search strategies, identify appropriate databases, access and use libraries and the internet, conduct electronic database searches, identify, retrieve, read [25] and appraise the literature [26–28, 32, 34, 86, 87, 102], synthesize evidence [41, 88, 102, 103], place findings within one’s local context [31], and utilize research findings [30] for best practices [43–45].
  Competency 2.5 – *Dissemination of research findings*: ability to share research findings with various stakeholders. This competency requires skills to summarize research findings, communicate and highlight key findings in a user-friendly way that may influence decision-making [30], develop a dissemination plan [71, 75, 83], write summaries, facilitate the production of knowledge synthesis documents [86, 91], distribute of relevant knowledge [29, 38], and evaluate the effectiveness of communication products [84].
  Competency 2.6 – *Use of research findings*: ability to apply research findings to clinical or policy decisions or to inform further research. This competency includes skills to interpret data and evidence [31, 42], apply research findings in ways that inform decision-making [32], formulate, evaluate and/or revise policies, procedures, protocols, client-specific programs and/or client standards of care [103], integrate evidence into practice with specific client populations [85, 104] and in their own setting [44], identify implications for one’s own practice [27, 29], and sustain interventions [30].
  Competency 2.7 – *Fostering innovation*: ability to use novel tools and strategies to improve practice or policy, address issues, assess and build service improvement approaches, and evaluate the impact of an innovation [41]. This competency includes targeted use of novel strategies and tools to reach different audiences [84], ability to integrate social media and online strategies [105], use models to guide practice and knowledge transfer activities within a certain context and bring about tangible improvements [38], and assess determinants of knowledge use [44].
  Competency 2.8 – *Knowledge brokering*: ability of applying KT strategies to facilitate the flow of knowledge, improve practice and policy [86] and increase research findings uptake [36, 98]. This competency includes applying techniques such as appreciative inquiry, conflict resolution, deliberative dialogue and negotiation, systems thinking, and adult learning processes [38, 86, 95]. The knowledge broker role relies on skills in scanning the environment for resources, conducting assessments to identify needs and readiness for change, developing strategies and planning change [38, 81, 87, 89, 92], facilitating knowledge exchange opportunities among various stakeholders in ongoing assessment of topic-specific issues and possible solutions [86, 87, 89, 93, 95, 102], guiding decision-makers in accessing, appraising, adapting and applying research findings [74, 80, 82, 85, 87, 89], and identifying opportunities for evidence to contribute to the policy cycle and to emerging research agendas [74, 95].
3. *KT competencies – Attitudes*
  Competency 3.1 – *Confidence*: a personal factor associated with belief in oneself and one’s abilities. Confidence is demonstrated by being self-assured, but not arrogant [45]. It requires attention to the political and value issues related to decision-making and control [30, 32, 38], and it is required in contributions to community partners [30] and in all relevant KT activities such as searching the literature, identifying relevant publications to answer clinical research questions, and critically appraising the literature [28].
  Competency 3.2 – *Having trust*: having to do with belief in the character, integrity, and truth of others. This was reported as an attitude essential for researchers, decision-makers [30], and policy makers [95] .
  Competency 3.3 – *Valuing research*: having a positive attitude toward research in practice [26], management and policy issues [74, 82], and valuing certain sources of research over others [32].
  Competency 3.4 – *Self-directed lifelong commitment to learning*: having an attitude that values experiential learning and persistence [38]. This competency is a valued attitude [26, 49, 85, 87] associated with a commitment to the development a culture of learning [31] and to continuous improvement [50]. It includes having a critical thinking attitude [32].
  Competency 3.5 – *Valuing teamwork*: having a positive attitude toward a culture of collective collaboration in research that is receptive to changing practice [27]. Enacting this competency supports the bridging of cultures and interests of various stakeholders to create high levels of engagement and commitment to knowledge exchange [95]. Individuals who value teamwork are comfortable and effective in dealing with people at all levels in various organizations [59], are committed to networking [76] and collaborative with a team-focused working style [66, 100, 105], and self-aware of their own abilities or limitations [32].

**Fig. 2:**
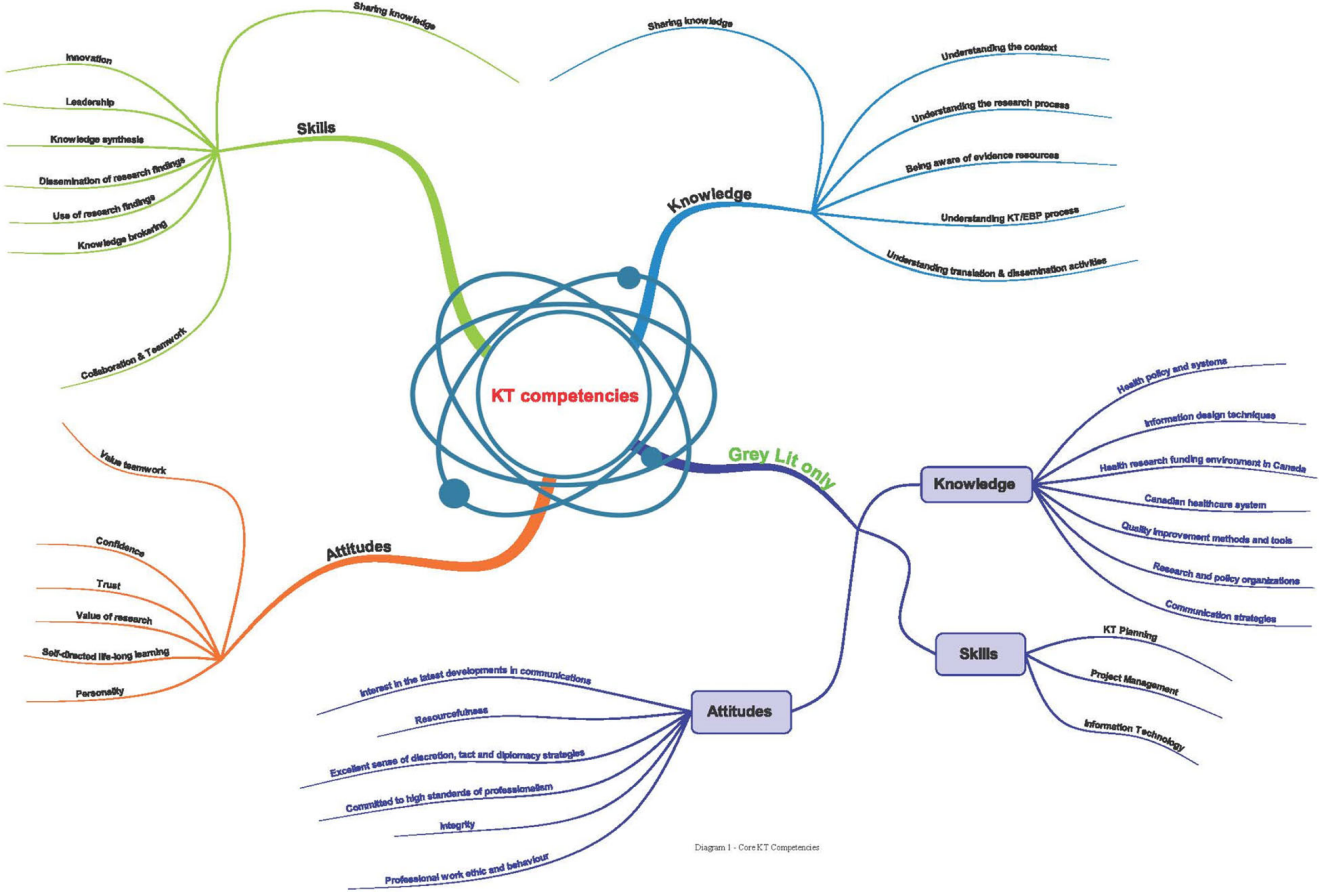
Core KT Competencies

The 19 core KT competencies described above are depicted in a conceptual diagram, using mind mapping, (Fig. 2), to illustrate the relationships among them and to inform the design of a future systematic review focused on interventions.

4. *“Other” KT competencies – Grey literature only*

Additional competencies were identified only in the grey literature and these involved knowledge and skills at the organizational, managerial, and leadership level related to abilities in communication, collaboration and adaptation. Themes of KT competencies that appeared only in the grey literature included

- *Knowledge* of quality improvement methods and tools [64], communication strategies [56], and health policy and systems [52].
- *Skills* related to KT planning [72, 86, 92, 96, 102], project management [75, 86], information technology use [76, 86, 87, 96, 102], sound judgment [66, 105], and discretion/tact/diplomacy and resourcefulness [105].

*Attitudes* such as integrity [42], commitment to professional work ethic and professional behaviour in interaction with internal and external contacts [48, 105], commitment to high standards of professionalism [52, 87], and interest in the developments in communications [105].

## Discussion

In this review, we identified, grouped and described 19 core KT competencies supported by both the academic and grey literature. We do not discuss the competencies found in the grey literature only, because they mainly referred to the organizational, managerial, and leadership level (not the scope or focus of our review) or they related to expectations in job descriptions (attitude-related competencies). The literature consistently acknowledges that KT is challenging [1]. We know that failing to use the best evidence may reduce the quality of care and result in poorer health outcomes for individuals and communities [106]. Thus, the core KT competencies can be used as a starting point for understanding the complexities of abilities one needs to be successful in this work. Each competency-category (knowledge, skills, attitudes) is discussed below followed by reflection on how this work may be applied. We also discuss the literature gaps, study strengths and limitations, and implications and suggestions.

### Discussion and reflection on KT competencies

*Knowledge* includes having an understanding of the *context* for KT activities, the research *process* and the basic elements for KT and EBP, and knowing the diverse *evidence* resources provided for these activities. These ideas are aligned with proposed KT frameworks on aging and health [11], and Scharff and associates’ [33] work on “KT and dissemination” that present a competency set in public health, which includes foundational knowledge competencies (e.g., understanding contexts, relationships, political/regulatory realities), understanding of KT, and dissemination. Knowing the context for KT activities and understanding organizational culture, values and behaviors toward research are both critical to success. For example, researchers should understand knowledge user contexts to successfully apply research findings in clinical environments or policy-making contexts [107]. Additional KT competencies found in the grey literature are also related to this notion of context. University courses usually emphasize understanding the research process and critically evaluating evidence; yet, often fail to give equal attention to the importance of understanding contexts and organizations’ political realities. Thus, these knowledge-related KT competencies may provide a basis for reflection on how professionals are prepared for KT in academic and organizational settings.

*Skills* refer to abilities in managing and leading teams in collaborative ways (e.g., knowledge brokering), and in sharing, synthesizing, disseminating and using research findings to foster innovation, improved quality, and effective health and health system outcomes. Scharff and associates’ [33] reported skill competencies including communication skills, and abilities to perform need assessments and implement change programs based on the assessed needs, develop marketing plans, use evaluation data from large datasets, and communicate to varying audiences in appropriate ways. The relevant literature in skills focused primarily on professionals at the individual level, implying that each professional should go through the process of asking clinical research questions, accessing the literature, identifying the most relevant publications, critically appraising them, synthesizing the best available evidence and applying the findings to practice. In our opinion, although all professionals and policy-makers should possess strong KT competencies, it is not feasible to expect that everyone goes through this entire process as part of every instance in their routine practice. Support of knowledge brokers or research facilitators to synthesize the evidence and facilitate the implementation process for clinicians and policy-makers is needed. Harvey and Kitson [108] described the concept of facilitation by referring to complex interventions that comprise the “active element of implementation” (p.6), that emphasize building and managing relationships among key stakeholders, and enabling others (instead of telling, teaching, persuading or coercing them) to act in a way that embraces processes for EBP. Organizations committed to KT in healthcare need to provide support and enable facilitation capabilities and skills to achieve implementation. Facilitators must be flexible and responsive, and able to apply a range of skills to support the implementation process and change, identify and negotiate barriers to EBP within different contexts, and tailor implementation of innovations and strategies to various settings and individuals involved [108]. We also note a recent shift to evidence-informed policy-making, that is based more on considerations (e.g., electoral considerations, public opinion, crisis management, personal preferences) than the best available evidence [109]. This understanding of ‘considerations’ emerges as an additional requirement for facilitation.

*Attitudes* include certain personal inclinations and values toward KT such as being confident, trusting and trustworthy, valuing research and teamwork, and aspiring to self-directed lifelong learning. The grey literature augments this list with additional competencies related to ascribing to a professional work ethic and being committed to high professional standards [42]. Attitude, a complex mental state that involves beliefs, feelings, and values [86], has been studied as an individual determinant. For example, attitude toward research as a personal characteristic is theoretically and empirically important and a predictive determinant of research utilization [110, 111] in various contexts [112]. However, the role of attitudes and beliefs toward research utilization is uncertain [111] suggesting that attitudes may have a strong inter-correlation with other determinants of research use such as professional role. Stetler and colleagues [113] identified attributes that facilitators need to develop to promote effective implementation of evidence into practice. Attitudes toward research use can be modified and may be the target of interventions through professional education processes [112]. While knowledge provides the foundation for carrying out the required skills, underlying attitudes may affect the way in which these skills are carried out. Regardless the limited research on the impact of positive attitudes in those supporting KT processes, many of these attitude-related competencies are cited as expectations in the job descriptions we reviewed. Therefore, from a competency-development perspective, a critical gap is the limited guidance in identifying ways to facilitate improvements in attitudes that will support KT.

### Role of personality

In addition to the above competencies, the literature presents personal characteristics or personality traits that cannot be listed as competencies per se, but nonetheless have been identified in both the academic and grey literature as being useful for individuals taking on a KT role and may have considerable importance in addition to learned competencies. These personality traits include being pragmatic and flexible [38, 45, 50, 54, 82, 85], positive [38, 54, 105], persuasive [74], entrepreneurial [87], proactive [61, 87], enthusiastic [85]; comfortable working in a dynamic environment [61], credible [76, 85], open-minded [61], autonomous [76], independent [49], self-sufficient and self-motivated [100], creative [50], and committed to principles of equity, inclusivity, respect and cultural competence [46]. Individuals with these personal traits value and reward flexibility [80], innovation and risk-taking [45, 87], and have high levels of imagination and inspiration [45]. We also noted that some of the characteristics identified as being important for KT were not attitudes (defined as opinions about or feelings toward something or someone), but they were actually personality traits (defined as factors that endure throughout a person’s life and growth and represent aspects of who that person is [114]. Thus, the characteristics such as “enthusiastic” or “agreeable” or “friendly” were taken as personality traits and labeled “personal attributes”.

#### Relationship of KT competencies to other competency statements

Many of these KT competencies have also been identified as components of researcher competencies [115]. This observation indicates that many competencies in the research process are a necessary part of KT competencies. Although research competencies overlap with KT competencies, the latter go beyond research competencies in that KT competencies also involve understanding of context, organization, practice and policy-making processes, and skills that are related to working with others, adapting research for individual settings, and applying research in practice and policy-making. In their seminal paper, Dawes and colleagues [116], while they did not refer to KT competencies, reported the competencies necessary for EBP that certainly overlap with those we identified as well. We submit that although similarities exist, the two constructs of EBP and KT are different. KT is broader and encompasses a more diverse range of activities aimed at increasing the use of health research evidence in practice, policy and subsequent research. Unlike EBP, KT activities occur at any point in the research cycle from the development of research evidence, to its implementation and evaluation in practice or policy settings. The fact that there are many terms associated with KT and the complexities of getting new knowledge into practice adds to the confusion. While standardization of terms or clarity through further research on the topic would be beneficial for inter-stakeholder communication [117], we recognize that this prospect would not be feasible in the near future.

#### Individual versus organizational competencies

We see a parallel between the work on KT competencies and the studies on quality of care and sources of “errors” [118]. Both individual and organizational practices contribute to health outcomes, such that focusing on individual behaviors or actions alone is not sufficient for understanding and reducing errors. For example, organizational context can improve safety outcomes, since there is an association between safety and organizational culture [119]. Even though our focus is on individual competencies, *organizational competencies* (e.g., assessing “organizational readiness” to adopt KT) are fundamental elements to successfully achieve KT activities; yet this literature was beyond the scope and focus of this review. To date, little research has been conducted on KT competencies at an organizational level and the identification of these competencies or organizational characteristics is certainly needed.

### Literature gaps

The first literature gap we identified was that most of the academic publications were about licensed or regulated healthcare professionals, followed by knowledge brokers. Other groups such as policy–makers were not addressed. Across all publications and resources, a scarcity of information existed on researcher KT competencies. Knowledge-based competencies were not always matched to skill-based competencies, and attitude-related competencies were sparse. Second, due to the infancy of this area of research, additional KT competencies may exist that have not been identified in this review. Finally, the empirical research was exploratory and descriptive. Clearly, research on KT competencies is limited; well-designed studies are essential to identify and test effective interventions and strategies to improve KT competencies, and to determine the impact of strong competencies on research use.

### Strengths and limitations

The use of robust and rigorous research methods and the identification of 19 core KT competencies comprise the strengths of this study. However, there are four limitations. First, the quality of the identified research was not assessed because it is beyond the mandate of a scoping review. Second, the search strategy may have missed some important publications due to our focus on identifying the keywords in titles and abstracts only. For example, the steps or action categories of a planned action model for change [120] may describe KT competencies. Third, the identified KT strategies and processes have been described or used primarily in the context of developed countries. Applications to other settings are unknown. Finally, the purpose of this synthesis was on KT competencies of individuals working in the health sector; therefore, we did not review organizational practices, processes or other contextual factors that may be required to support KT activities.

### Implications & Suggestions

Development of recommendations is not possible, because the primary sources for this synthesis were not critically appraised for methodological quality. Nonetheless, the recurring concepts identified across the publications provide initial foundational core KT competencies which offer implications (and suggestions) for education, practice, policy, knowledge brokering, and most certainly further research; they include

- Validation of the KT competencies in different settings for each of various stakeholder groups, including practitioners and the public (knowledge users), research facilitators (knowledge brokers), and researchers (knowledge producers).
- Identification or development and testing of targeted interventions and strategies to support competency development in each domain for stakeholder groups.
- Development of instruments for evaluating KT competencies. This work may help individuals and organizations identify competency gaps; develop targeted professional development plans to augment their effectiveness in KT roles, assess the effectiveness of professional development programs designed to address these gaps and quantify the impact, and include competency evaluation for determining the influence of specific active ingredients of KT interventions.
- Investigation of the effectiveness of the knowledge broker role as a facilitator of KT across health-related stakeholders, settings, and sectors and their impact on health and system outcomes. In a recent systematic review, when a facilitator supported clinical work, practitioners were 2.76 times more likely to adopt evidence-based clinical guidelines [121]; while the findings from another similar systematic review were unclear [106]. Adoption of a knowledge brokering approach may improve facilitation of resources, bring together health researchers and decision-makers, and develop a culture of evidence-based decision-making [15].

## Conclusions

The findings of this scoping review include 19 core KT competencies (addressing knowledge, skill and attitude domains) that overlap considerably with published research and EBP competencies. A sparse number of commonly used interventions and strategies were identified to enhance or develop KT competencies, and address research gaps that are required for individuals, teams, and health organizations. Identifying effective strategies is crucial to enable meaningful stakeholder engagement for potential changes in practice and improved care. Following this work, our primary suggestions refer to conducting rigorous studies on KT competencies and evaluate the ways these competencies contribute to KT success for each stakeholder group, while taking into consideration critical contextual factors.

## Additional files

Additional file 1: Literature search strategies. Electronic online databases, grey literature sources, search strategies and search terms, search concepts and terms (2 pages). (DOCX 50 kb)

Additional file 2: Description of the peer-reviewed/academic publications that are included in the KT scoping review (19 pages). (DOCX 78 kb)

Additional file 3: Theoretical Frameworks / Models / Theories. Description of theory, model or framework, the citing article, and application of the theory, model or framework (5 pages). (DOCX 64 kb)

Additional file 4: KT competencies found in the grey literature. Description of KT competencies (i.e., knowledge, skills, attitudes) found in the grey literature such as job description and other documents (22 pages). (DOCX 91 kb)

## Abbreviations

EBP: Evidence-based practice
KT: Knowledge Translation
MSFHR: Michael Smith Foundation for Health Research
