# Supplementary material for: Core knowledge translation competencies: a scoping review
Source: BMC Health Serv Res. 2018 Jun 27;18:502. doi: 10.1186/s12913-018-3314-4 (PMC6020388; doi:10.1186/s12913-018-3314-4)
Supplement: Supplementary file 1 — Literature search strategies. Electronic online databases, grey literature sources, search strategies and search terms, search concepts and terms (2 pages). (DOCX 50 kb) [file 12913_2018_3314_MOESM1_ESM.docx]

**Additional File 1** – Literature search strategies

Electronic online databases

1. PubMed / MEDLINE
2. Cumulative Index to Nursing and Allied Health Literature (CINAHL EBSCO)
3. Cochrane Library (Wiley)
4. EMBASE (Ovid)
5. NEOS Library Consortium Catalogue
6. Scopus
7. Theses Canada

Grey literature sources

Grey literature sources include websites of existing networks (e.g., InspireNet), relevant organizations (e.g., Canadian Institutes of Health Research [CIHR]; Canadian Foundation for Healthcare Improvement formerly Canadian Health Services Research Foundation [CHSRF]), conferences, government, non-governmental organizations, health research websites (e.g., National Collaborating Centre for Methods and Tools, World Health Organization), and databases specific to grey literature (e.g., KT Clearinghouse, Evidence-Informed Health Care Renewal portal). Specifically,

1. Evidence-Informed Healthcare Renewal (EIHR) Portal
2. INnovative health Services and Practice Informed by Research & Evaluation NETwork (InspireNet)
3. Grey Literature database
4. KT Clearinghouse
5. Center on Knowledge Translation for Disability and Rehabilitation Research (KTDRR) KT strategies’ database
6. Google (and other KT websites as discovered)
7. World Health Organization (WHO)
8. Working group and stakeholders input

A full list of grey literature resources are listed in our KT Pathways training documents available upon request (eventually it will be posted in the MSFHR website).

Search strategies and search terms

The conceptual building blocks for the search are outlined below. The actual search terms entered into databases and search engines included their variants (e.g. singular, plural, British/ American spelling, truncation) and were selected according to the characteristics and functionalities of the specific database. Detailed full search strategies are available upon request.

Search concepts and terms

| **KT Keywords**  **(combined with OR, searched in Title field)** | **AND** | **Competencies Keywords (combined with OR)** |
| --- | --- | --- |
| - knowledge transfer - knowledge utilization - knowledge use - knowledge translation - knowledge implementation - research in practice - knowledge mobilization - knowledge exchange - research transfer - research utilization - research use - research dissemination - knowledge dissemination - research exchange - research translation - knowledge to action - know do gap - evidence informed - diffusion of knowledge - research into practice - knowledge into practice - evidence into practice - translational science |  | - competence - competency - capacity building - skill - ability - training - curriculum - learning |
