# Supplementary material for: Core knowledge translation competencies: a scoping review
Source: BMC Health Serv Res. 2018 Jun 27;18:502. doi: 10.1186/s12913-018-3314-4 (PMC6020388; doi:10.1186/s12913-018-3314-4)
Supplement: Supplementary file 3 — Theoretical Frameworks / Models / Theories. Description of theory, model or framework, the citing article, and application of the theory, model or framework (5 pages). (DOCX 64 kb) [file 12913_2018_3314_MOESM3_ESM.docx]

**Additional File 3** – Theoretical Frameworks / Models / Theories

| **Theory, Model or Framework** | **Description** | **Citing Article(s)** | **Application** |
| --- | --- | --- | --- |
| **Theories and theoretical approaches** | | | |
| Diffusion of Innovations Theory (Rogers, 1995) | Adoption is driven by relative advantage, compatibility, complexity, trialability and observability of innovation | Bartelt, 2011 | Theoretical constructs (compatibility, observability and relative advantage) were evaluated as outcomes. |
|  |  | Kislov, 2014 | Diffusion of innovation is one knowledge domain related to KT competencies. |
| Dynamic Theory of Organizational Knowledge Creation (Nonaka, 1994; Nonaka & Takeuchi, 1999; Nonaka, Toyama & Byosiere, 2001; Nonaka & Von Krogh, 2009) | Describes the nature of knowledge as an asset within an organization, strategies for managing it, and the dynamic processes involved in the creation of both tacit and explicit knowledge and their subsequent interaction | Champagne, 2014 | Used to develop a logic model to describe and to evaluate the elements involved in knowledge creation. |
| Resource-based View of the Firm approach (Ambrosini & Bowman, 2009; Barreto, 2010; Eisenhardt & Martin, 2000) | Four orders of capabilities exist:   - Resources: Available factors owned or controlled by the organization - Ordinary capabilities: An ability to deploy resources to achieve simple knowledge mobilization (KM) tasks; - Core capabilities: The integration of resources and ordinary capabilities into complex KM projects aligned with an organization’s strategic directions; - Dynamic capabilities: An organization’s ability to generate, extend and modify these capabilities to enhance effectiveness and respond to change | Kislov, 2014 | KT-specific examples were generated for each order of capability from an organizational perspective; these orders inform recommendations for KT capacity building within organizations. |
| Individual and organizational capacity and learning as interconnected | Organizations are learning systems;  Individual learning is linked to organizational capacity building and learning | Kislov, 2014 | Used to advocate for a practice-based, multilevel approach to capacity building that targets both levels. |
|  |  | Champagne, 2014 | Used to develop a logic model to describe and to evaluate the elements involved in knowledge creation. |
| **Models** | | | |
| Iowa Model of Evidence Based Practice (Titler, 2001) | EBP involves a series of steps: Identify a need for EBP change; determine priority; form a team; gather evidence; appraise the evidence; decide whether to implement change; implement a pilot practice change and evaluate before full-scale implementation. Seeking evidence and solutions requires a team approach involving health professionals and organizations, in order to develop a culture of EBP. | Cadmus, 2008 | Justification for measuring personal and institutional barriers to research use, though explicit link to study design not described. |
| **Frameworks** | | | |
| Promoting Action on Research Implementation in Health Services (PARiHS) framework | Evidence, context and facilitation interact to influence the successful implementation of evidence based practices | Conklin, 2013 | Used to develop a logic model to describe and to evaluate the elements involved in knowledge creation. |
| Framework of Evaluative Inquiry as an Organizational Learning System (Cousins, Goh, Clark & Lee, 2004) | Organizational learning capacity and evaluation capacity are related to characteristics of the organization, evaluation activities and the consequences related to an organization’s capacity to learn, and the support structures (including evaluative inquiry) that sustain it. | Champagne, 2014 | Used to develop a logic model to describe and to evaluate the elements involved in knowledge creation. |
| Framework for the Analysis and Optimization of the Use of Scientific Evidence and Knowledge in Decision Making (Champagne, Lemieux-Charles & McGuire, 2004) | Critical factors in the analysis and optimization of evidence use include considering the processes used to generate knowledge, the characteristics of the evidence, the organizational and systemic context, the decision-makers, the decisions themselves, the manner in which the knowledge is used, the quality of the implementation, and non-evidence-related influences on complex decision-making. | Champagne, 2014 | Used to develop a logic model to describe and to evaluate the elements involved in knowledge creation. |
| Utilization-focused approach to evaluation | The focus of evaluation is on research utilization by all primary stakeholders; the process involves stakeholder participation in the development of evaluation methods and measures | Bowen, 2005 | Guided the development of evaluation measures and methods. |
|  |  | Champagne, 2014 | Used to develop a logic model to describe and to evaluate the elements involved in knowledge creation. |
| Translation and Dissemination framework (Brownson et. al., 2005) | Four stages to moving research into practice:   - Discovery – identifying determinants of disease/behaviours and testing intervention/service effectiveness - Translation – converting discovery into applicable forms for a range of stakeholders - Dissemination – spreading translated information to stakeholders - Change in health – as the result of change in behavioural, organization and environmental change, as well as program and policy adoption | Scharff, 2008 | Used to validate final list of competencies identified in the study. |
| Knowledge to Action Framework (Graham et al., 2007) | KT is a dynamic multidirectional process involving knowledge creation and refinement, as well as an action cycle with several steps to support the knowledge’s implementation (i.e. problem identification, barrier assessment, intervention selection and implementation, outcome and sustainability monitoring) | Wahabi, 2011 | Used as a KT framework during the workshop-based intervention; employed by participants in planning their case scenario KT interventions. |
|  |  | Illes, 2011 | Cited in background but not explicitly linked to study design/methods. |
|  |  | Bennett, 2016 | It was used to guide the introduction of KT to departments; increase KT itself within departments & clinical teams; and to facilitate KT becoming part of their department cultures. |
