# Supplementary material for: Core knowledge translation competencies: a scoping review
Source: BMC Health Serv Res. 2018 Jun 27;18:502. doi: 10.1186/s12913-018-3314-4 (PMC6020388; doi:10.1186/s12913-018-3314-4)
Supplement: Supplementary file 4 — KT competencies found in the grey literature. Description of KT competencies (i.e., knowledge, skills, attitudes) found in the grey literature such as job description and other documents (22 pages). (DOCX 91 kb) [file 12913_2018_3314_MOESM4_ESM.docx]

**Additional File 4** – KT competencies found in the grey literature

| **#** | **Publication ID** | **KT competencies (grey literature)** | | |
| --- | --- | --- | --- | --- |
|  |  | **Knowledge** | **Skills** | **Attitudes** |
| 1. **Job Description** | | | | |
| 1 | AFHTO, 2014  KTE Specialist | None | Skills to/in:   - work within a consultative model with demonstrated experience in working with diverse stakeholders at frontline, senior manager, community and provincial levels - group facilitation and adult education - successfully lead, organize and deliver projects, use effective project management methods, and optimize timely and complete delivery of concurrent projects within budget - establish and maintain positive relations with stakeholders - self-organize work with multiple deadlines and deliverables - work independently and as part of a team; - analyze, translate research and practice-based evidence, including experience with dissemination and education processes (online and in person) to impact utilization of the knowledge provided - performance measurement and improvement, using various types of data (e.g., survey, administrative). | None |
| 2 | Alberta SPOR SUPPORT Unit, 2015a  Program Coordinator – Knowledge Synthesis | Knowledge (understand):   - principles of knowledge synthesis | Skills to/in:   - building and maintaining partnerships with multiple stakeholders to achieve common goals - leadership and management to lead projects and people - work collaboratively in an environment characterised by ambiguity, shifting timelines and priorities, and multiple projects underway simultaneously - facilitation, collaboration, negotiation, and problem solving - systematic reviews | None |
| 3 | Alberta SPOR SUPPORT Unit, 2015b  Program Coordinator – KT & Implementation | Knowledge (understand):   - principles of Knowledge Translation and Implementation | Skills to/in:   - building and maintaining partnerships with multiple stakeholders to achieve common goals - coordinating large research projects, including proposal preparation, project coordination, and writing and editing scientific manuscripts - leadership and management to lead projects and people - working collaboratively in an environment characterized by ambiguity, shifting timelines and priorities, and multiple projects underway simultaneously - facilitation, collaboration, negotiation and problem solving | None |
| 4 | CAMH, 2015  Knowledge Broker | Knowledge (understand):   - knowledge exchange conceptual models, frameworks and strategies | Skills to/in:   - management of complex projects - facilitation of groups - adult education and training - development of work plans, budgets and communication plans - literature search, quality appraisal, applicability assessment and interpretation, and development of literature reviews/rapid reviews - a range of knowledge exchange methods (e.g. communities of practice, newsletters, workshops, online collaborative spaces, on-line repositories) - research, analyze and develop knowledge exchange options and strategies - project management, time management, interpersonal communication, and problem solving - collaborate well with team members with excellent networking, leadership, relationship building, interpersonal and listening skills to work effectively with a broad range of stakeholders and to facilitate the development of partnerships | - commitment to principles of equity, inclusivity, respect and cultural competence - self-directed |
| 5 | CCC, 2010  Knowledge Broker | Knowledge (understand):   - health care research and knowledge translation - the operation of The Cochrane Collaboration | Skills to/in:   - conducting a Cochrane systematic review - working with healthcare practitioners, government officials and patient/consumer groups - working with a variety of stakeholders to develop relationships and achieve shared goals - meeting and conference planning | None |
| 6 | CIHR-IGH, 2012  KT Manager | Knowledge (understand):   - unique needs of a range of KT audiences including policy makers, practitioners, researchers and consumers - health research and its funding environment in Canada | Skills to/in:   - developing and implementing knowledge translation strategies and products to meet needs of a diverse set of stakeholders, with particular emphasis on translating complex scientific data into suitable formats for various audiences; - effectively manage multiple tasks and priorities - program evaluation | - self-sufficient and self-motivated - collaborative, team-focused working style |
| 7 | CIHR-IGH, 2015  KT & Policy Manager | Knowledge (understand):   - unique needs of a range of KT audiences including policy makers, practitioners, researchers and consumers - health research and its funding environment in Canada | Skills to/in:   - knowledge translation theories and strategies - digital and print communications strategies - communication | - initiative - leadership - creativity - teamwork and cooperation - problem solving - strategic thinking - results orientation - relationship building - planning and organization |
| 8 | ICES, 2015  KT Officer | Knowledge (understand):   - in-depth knowledge of provincial and national health care, research and policy organizations | Skills to/in:   - designing and implementing community engagement initiatives - build and sustain positive and mutually supportive relationships, and anticipate and respond to their needs - read and understand scientific literature, including statistical information - translating research materials into lay language for both general and targeted audiences - public relations - manage multiple projects, workflows and timelines - social media competence | - Collaborative working style |
| 9 | McMaster-HF, 2015  Research associate, Scientific Lead, Evidence Synthesis | Knowledge (understand):   - health policy and systems | Skills to/in:   - conducting research syntheses including: developing search strategies; searching the literature; quality appraisal; synthesis; interpretation of findings; and writing up the results - management of activities across a team to deliver project outputs on time - preparing research protocol submissions for Research Ethics Boards - undertaking program evaluations | - commitment to high standards of professionalism |
| 10 | MSFHR, 2013a  KT Coordinator | Knowledge (understand):   - knowledge translation theory and methods | Skills to/in:   - knowledge translation - maintain confidentiality and deal with stressful, sensitive or difficult situations with tact and diplomacy, including ability to exercise judgment and discretion - conduct online web search/ literature review and analysis | - detail oriented |
| 11 | MSFHR, 2013b  KT Manager | Knowledge (understand):   - knowledge translation - management and budgeting - health research community, including an understanding of the research process as it relates to evidence-informed decision-making - the process and practice of research - health research resources currently available - project management - BC’s health system, particularly in understanding and working with challenges related to evidence-based decision making in the health sector | Skills to/in:   - interpersonal communication - identify key issues in complex situations - work in a collaborative manner with diverse groups - network thinking and problem solving - project management and knowledge translation - facilitating collaboration and partnerships in complex situations | - critical thinker experienced in synthesizing multiple streams of input - comfortable and effective in dealing with people at all levels in various organizations - comfortable working in a dynamic changing environment - enthusiastic - open-minded - proactive - part of an established network in the health sector (including academic, policy and research communities) - thrives in a team environment |
| 12 | MSFHR, 2015  KT Director | Knowledge (understand):   - knowledge translation - management and budgeting processes - health research community, including an understanding of the research process as it relates to evidence-informed decision-making - health research resources currently available | Skills to/in:   - strategic planning and project management - leadership - interpersonal communication - facilitation and negotiation skills - work in a collaborative manner with diverse groups - budgeting and financial management - comfort and expertise in presenting | - enthusiastic - open-minded - proactive - critical thinker able to identify key issues in complex situations |
| 13 | NCCAH, 2015  Research Associate | Knowledge (understand):   - Canadian public health environment which includes organizations, key players and how decisions are made in this environment - knowledge translation | Skills to/in:   - writing for the web and creating plain language documents - working in research and government environments work independently and also collaborate well with team members | None |
| 14 | Niagara Connects, 2013  Knowledge Broker, executive director | Knowledge (understand):   - KT theory and practice - community services - evidence-informed practice and decision making process - systems issues and stakeholders | Skills to/in:   - engage diverse audiences and build collaborations between groups and stakeholders - develop relationships and achieve shared goals - software and website content management systems (CMS) - designing, organizing, facilitating and evaluating knowledge exchange events such as meetings, webinars, conferences, community forums - Strong interpersonal, written and oral communication skills with great attention to detail - communicate using diplomacy and tact - strong critical thinking skills and ability to trouble shoot and problem solve | - professional work ethic and behaviours |
| 15 | OCE-CYMH, 2015 Knowledge Broker | Knowledge (understand):   - implementation science - knowledge exchange/ translation/ mobilization - evaluation and outcomes measurement | Skills to/in:   - work within a consultative model with demonstrated experience in working with diverse stakeholders at frontline, senior manager, community and provincial levels - group facilitation and adult education - build and maintain relationships with a broad range of internal and external stakeholders - tact and adaptability | - independent - self-directed |
| 16 | RHI, 2012  KT Specialist | Knowledge (understand):   - Canadian healthcare system - care - quality improvement methodology and tools | Skills to/in:   - communication - relationship building - problem solving | None |
| 17 | SHRTN, 2012  Knowledge Broker | Knowledge (understand):   - current health care or related research and KT concepts - evidence-informed practice and decision-making - healthcare system, market dynamics and priorities both regionally and provincially in systems of innovation | Skills to/in:   - work with diverse stakeholders (healthcare practitioners, researchers, gov officials, administrators, patient/consumers) - develop effective working relationships with others to achieve shared goals - collect and analyze data - define problems/problem solve - work with groups to problem solve, facilitate, participatory decision-making processes - use technology to facilitate networking/access to knowledge/dissemination - tailor evidence to diverse audiences - educate/train others in KT - marketing | - commitment to continuous improvement - creative - flexible |
| 18 | UofA-FofM&D, 2015  KT Program Coordinator | Knowledge (understand):   - principles of knowledge synthesis | Skills to/in:   - building and maintaining partnerships with multiple stakeholders to achieve common goals - lead projects and people - work collaboratively in an environment characterised by ambiguity, shifting timelines and priorities, and multiple projects underway simultaneously - facilitation, collaboration negotiation, and problem solving | None |
| 19 | UBC, 2012 Knowledge Broker | None | Skills to/in:   - community engagement and innovative knowledge translation approaches | None |
| 20 | UBC-CIHR-IGH, 2012  KT manager | Knowledge (understand):   - Advanced understanding of health research - Knowledge of the health research funding environment in Canada | Skills to/in:   - developing and implementing knowledge translation strategies and products to meet needs of a diverse set of stakeholders - Understanding of and sensitivity to a range of KT audiences including policy makers, practitioners, researchers and consumers - working in a government or university environment an asset - effectively manage multiple tasks and priorities - Exceptional oral and written communication abilities. | Attitudes:   - Self-sufficient and self-motivated - collaborative, team-focused working style |
| 21 | UBC-HELP, 2012  KT Director | Knowledge (understand):   - quantitative and qualitative research methodologies | Skills to/in:   - building leadership, community development policy and program development - lead change by creating a vision and taking appropriate action to ensure acceptance and support - strategic management and communications - work successfully with a wide range of groups and individuals, including community groups, researchers, the public sector and multiple levels of government | None |
| 22 | U of Guelph, 2014 Knowledge Mobilization (KMb) coordinator | None | Skills to/in:   - planning and delivering communication and knowledge mobilization strategies - project management in a dynamic environment with multiple priorities - web and social media content management - working with stakeholders at multiple levels - communication with multiple audiences - developing and delivering workshops | None |
| 23 | York U, 2012  KMb Officer | Knowledge (understand):   - knowledge translation theory and practice - service provider, researcher and policy maker perspectives - evidence-informed practice and decision making - information design techniques - systems issues and stakeholders | Skills to/in:   - build collaborations between groups and stakeholders - develop relationships and achieve shared goals - designing, organizing, facilitating and evaluating knowledge exchange events such as meetings, webinars, conferences, community forums developing knowledge products such as toolkits, newsletters, info-sheets and reports | - systems thinker - professional work ethic |
| 1. **Other** | | | | |
| 1 | CFHI, 2014 | None | Skills to/in:   - look for research in journals (that is by subscription, Internet, or library access) and non-journal reports (grey literature) by library, Internet access, or direct mailing from organizations - learn from peers through informal and formal networks - critical appraisal skills and tools for evaluating the quality of methodology and the reliability of specific research - determine relevance and applicability of research to the user context - communication skills to present research results concisely and in accessible language; to synthesize in one document all relevant research; link research results to key issues facing our decision makers | - value and reward flexibility, change, and continuous quality improvement |
| 2 | CHIN, 2013 | None | Skills to/in:   - evaluate and critique current practice against best available evidence/benchmarks - evaluate the need for practice improvement to promote safe, effective and reliable care that has a positive impact on client and system outcomes - uses systematic approaches to redesign care delivery to promote safe, effective and reliable care that has a positive impact on client and system outcomes - use research and outcome data to formulate, evaluate and/or revise policies, procedures, protocols, client-specific programs and client standards of care - use systematic approaches to retrieve, critically appraise, synthesize, apply and translate/ disseminate evidence and research knowledge into practical information for stakeholders - collaborate with clients and other providers | None |
| 3 | CHSRF, 2003 | Knowledge (understand):   - search out knowledge, synthesize research and scan for best practices, useful experiences, and examples from outside own organization - how to connect people to share and exchange knowledge; bring together people with common interests who rarely interact with each other | Skills to/in:   - evidence gathering; critical appraisal; mediation; communication; listening manage human intellect and help to put it to work - build networks - business skills, particularly marketing - act as advocate for the use of research-based evidence in decision-making and have a role in supporting and evaluating changes - bring researchers and decision makers together to exchange information and work together | - flexible - curious - well-informed on all aspects of a given sector - self-confident but not arrogant - imaginative - intuitive - inquisitive - inspirational leader - risk-taking |
| 4 | Graham, 2010 | Knowledge (understand):   - the rationale for and approaches to and methods of knowledge syntheses. | Skills to/in:   - complete knowledge syntheses and their funding proposals |  |
| 5 | Grimshaw, 2010 | Knowledge (understand):   - "What is QI?' and the differences/commonalities with KT research and with evaluation | None | None |
| 6 | Ciliska, 2012 | Knowledge (understand):   - the process of critical appraisal of an intervention study | Skills to/in:   - apply the criteria for critical appraisal of an intervention study and, where applicable, apply the intervention | None |
| 7 | CIHR, 2013 | Knowledge (understand):   - how social media can be used for KT | Skills to/in:   - using social media for KT - evaluate the effectiveness of a KT social media campaign | Interest in sharing research with and engaging users |
| 8 | CIHR, 2015 | Knowledge (understand):   - integrated and end-of-grant KT approaches | Skills to/in:   - develop integrated and end-of-grant KT plans | None |
| 9 | Fisher, 2010 | None | Skills to/in:   - act as information or knowledge intermediaries, concerned with enabling access to information from multiple sources and engaged in informing, aggregating, compiling and signaling information | None |
| 10 | Harris, 2010 | Knowledge (understand):   - knowledge translation, knowledge transfer and knowledge exchange frameworks, theories, models, mechanisms and strategies | Skills to/in:   - develop and maintain professional relationships between self and stakeholder organizations, policy/decision makers, researchers, care providers and networks, etc. - facilitate knowledge exchange between the audiences - appreciative inquiry, conflict resolution, deliberative dialogue, systems thinking, adult learning principles - foster partnerships between professionals, organizations and sectors - consult with knowledge users and producers on priority issues that affect care - review evidence (e.g., priority documents, literature reviews, position papers, etc.) - assess and analyse data from these multiple evidence sources (or facilitate the process to do this with others) | None |
| 11 | Harvey, 2013 | Knowledge (understand):   - how to develop a case study | Skills to/in:   - develop case studies as a tool for communicating with different audiences and stakeholders | None |
| 12 | Health Policy Project, 2014 | Knowledge (understand):   - policy issues that need to be adopted or changed - current data and research findings relevant to specific policy issues - the research process and a variety of methodologies (basic understanding) - policy processes, key influences, and current policy priorities - barriers to use of data and research - policy makers' information needs | Skills to/in:   - collaborate with researchers to identify data and research findings relevant to the policy process - extract, interpret, summarize, and translate data into non-technical language - work with journalists and others who disseminate information to reach decision-makers - effectively communicate research findings and evidence-based policy recommendations through print, digital and verbal formats, interpersonal communication, media outlets, constructive engagement with policymakers and decision-makers, or public events - communicate data needs back to researchers - facilitate participatory exchanges between researchers, technical experts, policy champions, coalitions/ networks, and other stakeholders - evaluate the effectiveness of knowledge translation efforts to determine their contributions to policy outcomes and the lessons learned to improve future activities | - objective and committed to the use of data and research for policymaking - committed to eliminating jargon and technical language so that research can be understood by non-technical audiences - persuasive |
| 13 | HSF, 2014 | Knowledge (understand):   - what a KT plan is - what barriers and facilitators are - that planned KTE activities contribute to the relevance, feasibility, and anticipated application of the research results to policy, practice, and health outcomes | Skills to/in:   - engage end users in developing KT plan - developing the most effective KT approach to optimize use of the research - identifying potential barriers and how to overcome them in implementing the KT plan | None |
| 14 | Lamari, 2014 | Knowledge (understand):   - how to assess new knowledge e.g., methodology and findings of research - how to synthesize findings | Skills to/in:  managing social media   - monitoring and acquisition of knowledge - systematically synthesize texts introducing new knowledge judged to be meaningful - facilitator of development to “educate,” disseminate, explain complex, useful knowledge in simple terms, clarify or influence the decision making of users of this new knowledge | None |
| 15 | Levesque, 2014 | Knowledge (understand):   - knowledge brokering - what is knowledge - the best sources of synthesized evidence and original studies within their content area | Skills to/in:   - link researchers and decision makers, facilitating their interaction so that they are able to better understand each other's goals and professional culture, influence each other's work, forge new partnerships, and use research-based evidence | - inquisitive - enthusiastic - flexible - inspirational - imaginative - highly credible - keenly interested in learning |
| 16 | Melaniebarwick.com, 2008 | Knowledge (understand):  use of a template in planning KT activities | Skills to/in:   - use a template in planning KT activities, ensuring partners, expertise, users, messages, goals, strategies, process, evaluation, resources, budget and implementation are identified | - None |
| 17 | NHSScotland, 2012 | Knowledge (understand):   - different forms of knowledge, including explicit and tacit knowledge, “know-what”, “know-how”, “know-who” - the principles of co-creation and co-production as they relate to KT - the purpose of each type of knowledge, how each is generated and validated, the differences between knowledge and evidence, and how each type of knowledge contributes to planning and decision-making to improve quality of health and care   the difference between local and generalizable knowledge | Skills to/in:   - apply improvement principles and tools to design, implement and measure impact of tests of change or other improvement methods - apply appropriate measurement techniques to assess impact of initiatives on awareness, engagement, user satisfaction, decisions, practice and ultimately service needs and outcomes - exert influencing and negotiation skills to engage clinical, senior management and practitioner engagement in implementation - lead and manage change in use of knowledge in practice, policy and planning in complex organisations - plan and implement strategy to embed knowledge support in the work of frontline practitioners - champion and promote at all levels in the organisation the application of knowledge to improve quality of health and care - contribute to and encourage ideas and innovation in facilitating the application of knowledge to improve quality of health and care - demonstrate collaborative, distributed leadership, pedagogical approaches to develop workforce capabilities in translating knowledge into action mechanisms of supporting learning including web-based learning, virtual classrooms, social networking, face to face  training, digital collaboration, individual and group reflection - identify opportunities to engage partners, including service users, in developing services and resources; enquiry techniques – e.g. interview, focus group, questionnaire, observation, listening and responding, concept mapping - to define real - as opposed to perceived - knowledge needs | - collaborative leadership entrepreneurial approach - solution-focused mind-set - innovating - proactive approach - demonstrating professionalism - customer / User-centred approach - commitment to continuous learning |
| 18 | Opsahl, 2010 | Knowledge (understand):  what a dissemination plan is | Skills to/in:   - develop a dissemination plan as a key component of initial research designs - use innovative strategies and tools for designing interventions to reach different target audiences | - None |
| 19 | Parry, 2015 | Knowledge (understand):   - open access |  | - None |
| 20 | PHAC, 2008 | Knowledge (understand):  Knowledge of the specific context/field | Skills to/in:   - apply knowledge to inform health policies and programs - summarize key findings from a report to support policy changes | - None |
| 21 | PHRIS, 2006 | Knowledge (understand):   - how to make connections between groups of people to share and exchange knowledge for policy making - policy development and implementation - awareness of the systems of policy making and government - communities of practice and knowledge exchange networks | Skills to/in:   - identify key research messages and target audiences - build relationships and networks - identify opportunities for the evidence to contribute to the policy cycle   provide research summaries in ordinary language develop workshops and training programs/sessions | - charismatic - knowledgeable - highly trusted - scientific credibility - authority - impartial stance in a political context |
| 22 | Reardon, 2006 | Knowledge (understand):   - understand the importance of having an ongoing relationship with public policy-makers | Skills to/in:   - developing a relationship with public policy makers | - Interests in building relationships with public-policy makers |
| 23 | Roxborough, 2009 | None | Skills to/in:   - creating networks of researchers and decision-makers - promoting evidence use - transforming clinical or management questions into research questions - helping researchers and decision-makers establish priorities - guiding decision-makers in accessing, appraising, adapting and applying research evidence - helping decision-makers find, develop, or commission synthesized research and develop tailored | - inquisitive - enthusiastic - flexible - inspirational - imaginative - highly credible - keenly interested in learning |
| 24 | SickKids Foundation, 2008 | Knowledge (understand):   - key factors and considerations (KT goals, collaboration, research stage, participants, methods, and resources and implementation) in developing a KT plan | Skills to/in:   - developing KT plans including goals, approaches for collaboration with users in the research stage, determining the best KT approach, estimating KT plan resources and implementation of KT plans | None |
| 25 | UofC-CRISP, 2012 | Knowledge (understand):   - Understand the various D&I models | None | None |
| 26 | UofGuelph, 2015 | Knowledge (understand):   - the processes involved in planning, implementing, and evaluating KMb strategies | Skills to/in:   - assess the quality of evidence - create a KMb plan to inform or raise awareness - create strategies to collect, collate and package evidence in a way that makes it accessible and relevant to users - select appropriate tools and techniques to monitor and evaluate the success of KMb information sharing and awareness-raising efforts - conduct stakeholder analyses to understand needs of target audiences, interest in and capacity to engage with the evidence - recognize individual, organizational and system-level barriers and enablers to using and applying evidence - convening models (such as workshops and communities of practice) and facilitation techniques to support engagement with and dialogue around evidence - determine the various roles of KMb partners and practitioners in enhancing user engagement - select appropriate tools and techniques to evaluate the success of KMb engagement efforts | None |
| 27 | UofT-CRWH, 2009 | Knowledge (understand):   - the importance of building relationships with stakeholders - building opportunities for interaction into the research process - sharing work in ways that are meaningful and accessible to stakeholders | None | None |
| 28 | Ward, 2010 | Knowledge (understand):   - a model of the knowledge transfer process - analysing the context which surrounds the producers and users   knowledge information management, linkage, capacity building, decision and implementation support | Skills to/in:   - describe the processes involved in planning, implementing, and evaluating KMb strategies - assess the quality of evidence from a variety of sources and disciplines - recognize the contexts in which knowledge is created, validated, and used - identify the barriers and enablers to using and sharing evidence in different contexts and among different target audiences - create a KMb plan to inform or raise awareness - conduct stakeholder analyses to understand needs of target audiences, interest in and capacity to engage with the evidence - recognize individual, organizational and system-level barriers and enablers to using and applying evidence - review various convening models (such as workshops and communities of practice) and facilitation techniques to support engagement with and dialogue around evidence | None |
| 29 | WHO, 2006 | Knowledge (understand):   - knowledge mapping - knowledge value chains (focusing on the processes of knowledge acquisition, creation, sharing/dissemination, utilisation/application, and performance assessments within the context of the strategic goals of the health system - diffusion of innovation in clinical practice - health service management and organizational learning - strategic advocacy - community mobilization and social entrepreneurship - knowledge brokering | Skills to/in:   - improve access to evidence for policymakers - link policymakers and researchers - access, synthesize, package and communicate evidence for policy and practice and for policy-relevant research agenda - translate new knowledge to other contexts - link research to action - develop synthesis reports and summaries of evidence in response to questions raised by stakeholders | None |
