# Supplementary material for: Core knowledge translation competencies: a scoping review
Source: BMC Health Serv Res. 2018 Jun 27;18:502. doi: 10.1186/s12913-018-3314-4 (PMC6020388; doi:10.1186/s12913-018-3314-4)
Supplement: Supplementary file 2 — Description of the peer-reviewed/academic publications that are included in the KT scoping review (19 pages). (DOCX 78 kb) [file 12913_2018_3314_MOESM2_ESM.docx]

**Additional File 2** – Description of the peer-reviewed/academic publications included in the KT scoping review

| **#** | **1^st^ Author’s Name**  **& Publication Year** | **Population Studied** | **Study Design** | **KT Competencies** | | | **Interventions/Strategies**  **to improve KT comps** |
| --- | --- | --- | --- | --- | --- | --- | --- |
|  |  |  |  | **Knowledge** | **Skills** | **Attitudes** |  |
| A. **Empirical** | | | | | | | |
| **1.** | Barratt, 2016 | 35 healthcare and public health organizations in London and South East England. | Descriptive cross-sectional (surveys) | - Research methods including data analysis - Evaluation techniques - Understanding development in research | - Time management - Applying research to practice | None | None |
| **2.** | Bartelt, 2011 | 486 paediatric health care professionals. | Descriptive cross-sectional (surveys) | - Understanding of organization or structure of electronic library databases | Skills in using resources to access EBP information such as:   - searching databases - critiquing and/or synthesizing the literature - computer skills - reading peer-reviewed articles | None | Formal and continuing education (EBP educational series were provided). |
| **3.** | Bennet, 2016 | 60 occupational therapists working at a large metropolitan hospital. | Participatory action research | - Knowledge of KT use - Change management - Understanding types of KT interventions | - Teamwork - Communication on KT language - Ability to enact a structured KTA process | Sense of optimism about KT impacting clinical outcomes. | None |
| **4.** | Bowen, 2005 | 62 (101 interviews); Team members from partner organizations involved in The Need to Know Project in support of rural decision-making by regional health authorities; Project's Advisory Committee & CEOs. | Participatory Action Research (semi-structured interviews) | - Knowing 'how things really work' in the context of knowledge users - Understanding the research and its importance - Knowledge of the importance of: - creation of an environment of interest in, and openness to, research - opportunities for collaborative research - a shared vocabulary and conceptual base - a forum for sharing - understanding of research findings - understanding of implications for practice - application and utilization of research - sustainability of interventions | Skills (ability to/in):   - communicate findings in a manner that influences decision-making/more user-friendly way - conduct research of relevance to intended users - develop authentic, respectful, effective and collaborative peer/working relationships - facilitation - adult education - networking & communication | - Confidence in contributions of community partners - Being nice - Attention to the political and value issues related to decision-making and control (the ‘personal factor’) - Trust between researchers and community partners and decision-makers | Organizational support such as time to review studies, dialogue between administration and staff; used participatory evaluation to explore characteristics of KT. |
| **5.** | Cadmus, 2008 | 987 acute and specialty care hospital-based registered nurses | Descriptive, exploratory survey | Knowledge of:   - evidence resources and how they work - research   Understanding:   - databases - research articles - use of evidence-based tools | Skills (ability to):   - identify, access, and evaluate information - conduct a clinical database search, appraisal, synthesis, computer skills - understand databases - understand research articles - understand the use of evidence-based tools | - value research in practice - value of research   self-directed lifelong learning | Formal and continuing education. It was provided information provided at the bedside, dialogue between administration and staff, time to review and implement research findings, colleague support, creation of a culture where EBP is valued and expected; training to use databases; management support to access databases; research/EBP committees; provision of evidence-based policies/procedures; on-unit support by librarian. |
| **6.** | Champagne, 2014 | Executive Training for Research Application (EXTRA) and SEARCH Canada Program fellows, colleagues, supervisors, Vice Presidents and CEOs (n=84). | Analysis of six case studies at the individual, group and organization level (triple comparative). | Knowledge in EIDM for decision-makers | Skills (ability):   - for sound management and leadership - in conducting and using research (more emphasis on management and leadership in the skills) - to motivate others toward the use of EIDM - to acquire (look for and access research) - to assess the quality and relevance of research - to adapt (i.e., summarize and relate research to context) - to apply (i.e., how research recommendations inform decision making) | - culture of learning - leaders: commitment to the development of research capacity and utilization in their organizations - attitudes toward EIDM | Involvement of people in an organization, as well as high quality, active communication seems to be essential for organizational changes to occur. |
| **7.** | Conklin, 2013 | Knowledge brokers in a Seniors’ Health research transfer network. | Description of a mixed-methods evaluation of the KB program development: focus groups, interviews, review of documents meeting notes, job descriptions | None | Skills (ability to/for):   - use models from the peer-reviewed literature to guide practice - focus on using knowledge transfer to bring tangible improvements - place knowledge transfer activities within the context of a performance improvement model that will help focus resources on closing gaps - allow for interaction through enabling technology - work with teams and to develop relevant models and approaches - foster relationships and creating operational groups capable of producing tangible results - ensure the ongoing health and success of network alliances - link researchers who produce scientific knowledge and practitioners who produce experience-based knowledge with knowledge users - establish trusting relationships that encourage conversations about the introduction of changes into frontline practices - implement a strategy for forming partnerships and engaging stakeholders - interpersonal communication - find, appraise, transform, and disseminate relevant knowledge - identify knowledge sources - assess the usefulness of knowledge and adapting it for local contexts - synthesize knowledge into usable formats - find meaningful roles for knowledge users to play in the research process - facilitate social interactions and collaborative processes - facilitate skill development and adult education - disseminate information to CoP participants - facilitate discussions and meetings - handle logistical setup for meetings - prepare materials for presentations - troubleshoot technical problems - assist with membership growth - help with the preparation of knowledge translation tools - engage in analytical and planning activities - coach and support people - expand existing networks - translate knowledge so it can be used by specific groups - handle administrative functions - direct, support, and negotiate - subject matter and technical skills (e.g., learning) - personal qualities that equip them to work in ambiguous social environments (including time management, flexibility, and persistence) - scanning the environment for resources - conducting assessments to identify needs and readiness for change - developing strategies and plans to bring about change - sensitivity to organizational and occupational culture as barriers to or enablers for change - promote mutual understanding - develop new capacity - technology - working with people and groups - collaborate - research - influence and persuade - verbal and written communication - project management - empower others - technical skills | - be pragmatic and positive - flexible - self-confident - experiential learner   persistent | Literature review on knowledge brokering. |
| **8.** | Fairbrother, 2014 | 169 nurses and midwives in a Wales health district, excluding those in advanced practice, management or education roles. | Quantitative, descriptive survey (exploratory). | Knowledge of:   - how to find organizational info   how to find appropriate research reports | Skills (ability to):   - find research evidence - find organizational info - use the library - use the internet to search - review research evidence - review organization info - use research to change practice - use organizational info to change practice - understand research reports - identify implications of organizational info for own practice - confidently judge quality of research reports | - culture of team receptive to changing practice | Evaluated self-reported skill levels in KT. |
| **9.** | Jessani, 2016 | 12 academics and faculty leadership (identified as KBs) from six SPHs in Kenya; and 11 national health policy-makers with whom they interact. | Qualitative (interviews) | - Applied Research - Polices impacting health care - Knowing economic impact of recommendations to practice | - Leadership skills - Interactive skills – ability to form relationships - Communication skills | - Having a moral and social conscience - Being committed and determined - Having respect for others | None |
| **10.** | Lundgren, 2013 | 42 nurses who completed bachelor thesis during their education and had 1-1.5 year work experience post-graduation. | Qualitative | Knowledge of:  application of research findings | Skills (ability to):   - find and evaluate research findings - critically think   apply research findings | - more analytical and selective regarding research sources - critical thinking attitude - increased confidence in own abilities - increased self-awareness of own abilities or limitations in collaborative situations | Formal and continuing education. Evaluated the bachelor’s thesis as a tool to enhance KT knowledge and its application to practice. |
| **11.** | Scharff, 2008 | Academics, and public health professionals from health departments, community-based agencies and healthcare delivery organizations (Delphi: 22;  focus groups: 25). | Delphi method; focus group, work group review of developed competencies | The final set of 24 translation and dissemination (T&D) competencies were developed in three groups:  1. Foundational (n=6)   - understand contextual environments - understand relationships and bases of power within & across organizations and communities - understand how research is translated into the policy process - understanding the political and regulatory realities   2. Translational & disseminational (n=11)  3. Change (n=7) | Skills (ability to/for):  Foundational:   - conducting needs assessments - identifying existing, effective evidence-based programs & materials   Translation and dissemination:   - tailor language, style, delivery modalities, and communication channels to specific audiences - develop or modify existing evidence-based programs to specific contexts so they are culturally and linguistically appropriate - use a variety of communication and dissemination methodologies - ability to communicate large and complex datasets that are understandable to target audiences - recognize and use a variety of communication forms including non-verbal and visual - create processes of communication that allow systematic input and feedback from audiences - develop marketing plan to promote evidence-based interventions to communities - communicate characteristics of public health interventions that are associated with widespread adoption and maintenance - use innovative methods (quantitative/qualitative) to evaluate and implement translation & dissemination activities - develop processes for dissemination - use innovative methods to communicate lessons learned   Change:   - identify and effectively collaborate and create linkages with stakeholders - engage in communication to reduce stakeholder resistance to change - identify and implement strategies to enhance program adoption and sustainability - provide stakeholder support for adoption and sustainability of programs and policies - recognize the balance between research and community needs - provide capacity building for finances, organizational structure and processes, and training infrastructure - use a variety of policy strategies (advocacy, policy briefs, lobbying) to effectively influence change | None | Descriptive research to document competencies |
| **12.** | Tabak, 2017 | Researchers, practitioners, and policymakers participated in 2014 -2015. | Concept mapping | Research process | - Communication skills - Practice partnerships - Understanding multi-level context - Make research relevant & meaningful | None | None |
| **13.** | Wahabi, 2011 | 21 family medicine consultants as clinical tutors and trainers for a Family Medicine diploma program. | Unclear | Competencies are implied by the content of the teaching and the evaluation of learning and include  Knowledge of:   - how to access literature - critically appraise literature - rank data according to hierarchy of evidence | Competencies implied Skills (ability to):   - apply data and evidence to local and cultural contexts - access literature - critically appraise literature - rank data according to hierarchy of evidence | None | Evaluated two teaching models (KT project and debates) to teach KT. |
| **14.** | Walker, 2014 | 584 chiropractors registered in Australia | Survey research | Knowledge (understand):   - EBP process - form a research question - develop search strategies - identify the most appropriate databases on the topic under investigation - confidently and critically appraise the literature | See previous column on Knowledge survey items & on Skills (ability to):   - use literature to make clinical decisions - use databases to find literature - critically appraise literature | Survey items on Attitudes:   - attention to literature | Conducted a survey of chiropractors on KT and EBP. |
| **15.** | Yost, 2014 | 51 attendees at the 2010 EIDM workshop (nursing, public health, library services, and nursing education). | Explanatory mixed methods, longitudinal. | None | The tool these researchers developed included the following Skills (ability to):   - formulate research (clinical) questions - search for evidence - critically appraise literature - interpret literature and   determine relevance for practice | None | Evaluated the impact of a KT workshop. |
| B. **Theoretical/Conceptual** | | | | | | | |
| **1.** | Castiglione, 2012 | n/a | n/a | n/a | n/a | n/a | n/a |
| **2.** | CHSRF, 2003 | n/a | n/a | n/a | n/a | n/a | n/a |
| **3.** | Grimshaw, 2012 | n/a | n/a | n/a | n/a | n/a | n/a |
| **4.** | Kislov, 2014 | n/a | n/a | n/a | n/a | n/a | n/a |
| **5.** | Straus, 2009a | n/a | n/a | n/a | n/a | n/a | n/a |
| **6.** | Straus, 2011 | n/a | n/a | n/a | n/a | n/a | n/a |
